# Document-Level Biomedical Relation Extraction Leveraging Pretrained Self-Attention Structure and Entity Replacement: Algorithm and Pretreatment Method Validation Study

**DOI:** 10.2196/17644

**Published:** 2020-05-29

**Authors:** Xiaofeng Liu, Jianye Fan, Shoubin Dong

**Affiliations:** 1 Communication and Computer Network Key Laboratory of Guangdong School of Computer Science and Engineering South China University of Technology Guangzhou China

**Keywords:** self-attention, document-level, relation extraction, biomedical entity pretreatment

## Abstract

**Background:**

The most current methods applied for intrasentence relation extraction in the biomedical literature are inadequate for document-level relation extraction, in which the relationship may cross sentence boundaries. Hence, some approaches have been proposed to extract relations by splitting the document-level datasets through heuristic rules and learning methods. However, these approaches may introduce additional noise and do not really solve the problem of intersentence relation extraction. It is challenging to avoid noise and extract cross-sentence relations.

**Objective:**

This study aimed to avoid errors by dividing the document-level dataset, verify that a self-attention structure can extract biomedical relations in a document with long-distance dependencies and complex semantics, and discuss the relative benefits of different entity pretreatment methods for biomedical relation extraction.

**Methods:**

This paper proposes a new data preprocessing method and attempts to apply a pretrained self-attention structure for document biomedical relation extraction with an entity replacement method to capture very long-distance dependencies and complex semantics.

**Results:**

Compared with state-of-the-art approaches, our method greatly improved the precision. The results show that our approach increases the F1 value, compared with state-of-the-art methods. Through experiments of biomedical entity pretreatments, we found that a model using an entity replacement method can improve performance.

**Conclusions:**

When considering all target entity pairs as a whole in the document-level dataset, a pretrained self-attention structure is suitable to capture very long-distance dependencies and learn the textual context and complicated semantics. A replacement method for biomedical entities is conducive to biomedical relation extraction, especially to document-level relation extraction.

## Introduction

A large number of biomedical entity relations exist in the biomedical literature. It is beneficial for the development of biomedical fields to automatically and accurately extract these relations and form structured knowledge. Some biomedical datasets have been proposed for extracting biomedical relations, such as drug-drug interactions (DDI) [1], chemical-protein relations (CPR) [2], and chemical-induced diseases (CID) [3]. The former 2 datasets are sentence-level annotated datasets that extract relations on a single sentence containing a single entity-pair mention, and the latter is a document-level annotated dataset, which means that it is uncertain whether relations are asserted from within sentences or across sentence boundaries.

Most approaches [4-7] have focused on single sentences containing biomedical relations. For example, Zhang et al [4] presented a hierarchical recurrent neural network (RNN) to combine raw sentences with their short dependency paths for a DDI task. To deal with long and complicated sentences, Sun et al [5] separated sequences into short context subsequences and proposed a hierarchical recurrent convolutional neural network (CNN). Because these approaches cannot be directly applied to document-level datasets, some existing methods [8,9] divided the document-level dataset into 2 parts and trained an intrasentence model and an intersentence model. Nevertheless, because of long-distance dependencies and co-references, their methods cannot be adapted to cross-sentence relation extraction. Furthermore, splitting the dataset resulted in noise and rule-based mistakes.

Currently, for intersentence relation extraction, some studies [10-12] generate dependency syntax trees within sentences and across sentences and employ a graph neural network to capture dependencies. However, it is costly to build dependency syntax trees. In addition, few studies, except those by Li et al [13] and Verga et al [14], have considered the influence of noisy data due to the segmentation of datasets and taking advantage of the textual context. For a document-level annotated corpus, an entity-pair mention within sentences or across sentences has a biomedical relationship by thinking simply, which will undoubtedly cause errors and may ignore plenty of useful information such that many sentences with co-occurring or co-referential medical entity mentions refer to biomedical relations.

For example, the chemical-disease relation (CDR) dataset is a document-level corpus designed to extract CID relations from biomedical literature [15]. For CID relation extraction, most current methods [8,16,17] divide the CDR dataset into intrasentence-level and intersentence-level relation instances using heuristic rules. Although these heuristic rules are effective, they inevitably generate noisy instances of CID relations or ignore some useful information. For example, the following sentence expresses CID relations between the chemical amitriptyline and the disease blurred vision: “The overall incidence of side effects and the frequency and severity of blurred vision, dry mouth, and drowsiness were significantly less with dothiepin than with amitriptyline.”

According to heuristic rules [8], the token distance between two mentions in an intrasentence level instance should be <10. The token distance between the chemical amitriptyline and the disease blurred vision in this example is 12; therefore, this sentence is discarded. However, factually, this sentence is the only sentence in the document [18] that describes the CID relation between the chemical amitriptyline and the disease blurred vision. Obviously, heuristic rules cannot precisely partition the CDR dataset, and they can induce the wrong classification by models, although they use multi-instance learning to reduce these errors.

Therefore, when constructing relation instances from a document-level dataset, it is necessary to consider sentences with multiple mentions of target entities in the entire document. While treating all target entities in a document as a whole brings benefits, the challenges are very long-distance dependencies and complex semantics, from which traditional neural networks such as CNN or RNN cannot accurately extract document-level relations. Recently, pretrained self-attention structures, such as SciBERT [19] and BERT [20], were proposed and were not necessarily better than RNN at capturing long-range dependencies. However, they performed better at increasing the number of attention heads [21]. A pretrained transformer has already learned more semantic features, and it performs well for sentence-level relation extraction; however, it did not apply to document-level relation extraction.

To address these problems, this paper proposes a pretrained self-attention mechanism and entity replacement method to extract document-level relationships. In this way, this paper has several contributions. First, to avoid errors by dividing the document-level dataset, this paper proposes a new data preprocessing method that treats the target entity pair of some sentences in a document as an instance. Second, to better focus on the target entity pairs and their context, a replacement method is proposed to replace biomedical entity pairs with uniform words. Compared with the different entity preprocessing for biomedical entity pairs, the replacement method is more effective for biomedical relation extraction. Third, to solve the problem of long-distance dependencies and learning complex semantics, a pretrained self-attention structure is proposed for document-level relation extraction and to achieve superior performance than state-of-the-art approaches. Through analysis and visualization of the model structure, the effectiveness of the self-attention structure for document-level datasets is demonstrated.

## Methods

### Data Preprocessing for the Document-Level Corpus

As already mentioned, splitting the document-level corpus will increase noise and may lose some useful information. To address this problem, the sentences in which the target entity pair is located and the sentences between them are constructed to an instance. This approach has the following benefits. First, it does not introduce error messages. The sentences do not need to be labeled after the segmentation of the dataset. The relationship between the marked relation pairs in the document corresponds to the instances one by one. Second, it discards useless information that is not related to the relationship of the target entities. Some are not related to those sentences in which the target entities are located; hence, they are noise for relation extraction. Discarding them will focus the model on the sentences in which the entity pair is located. Third, it keeps a lot of useful information, such as contextual information about entities and the relationship of entities.

**Figure 1:**
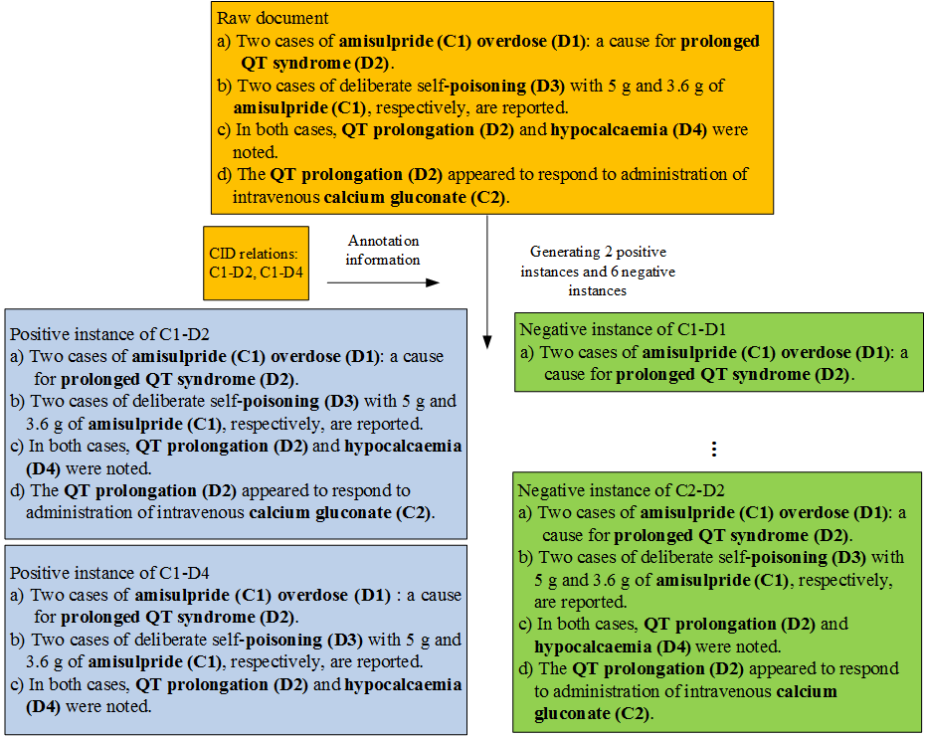
An example of document-level relation instance construction.

As shown in Figure 1, a document [22] in the CDR dataset is constructed into biomedical relation instances. All chemical or disease entities are bold.

In this document, there are 2 chemical entities, “amisulpride” (C1) and “calcium gluconate” (C2), and 4 disease entities: “overdose” (D1), “prolonged QT syndrome/QT prolongation” (D2), “poisoning” (D3), and “hypocalcemia” (D4). It should be noted that C1, C2, D1, D2, D3, and D4 are added to the document to indicate which are chemical entities and which are disease entities. Hence, the document can be constructed into 8 instances, in which 2 instances of C1 and D2 or C1 and D4 have CID relations. a), b), c), and d) conformed the instance of C1 and D2. a), b), and c) conformed the instance of C1 and D4.

Semantically, both the intralevel sentence a) and the interlevel sentences b) and c) express the CID relationship of C1 and D2. However, according to heuristic rules, b) and c) will be discarded because only the entities that are not involved in any intrasentence level instance are considered at the intersentence level. Third, instances are full of contextual information of chemical and disease entities, which is conducive to document-level relation extraction when exploiting it well.

There are lots of biomedical entities in a document. When constructing the instances of the target entity pair, it is inevitable that the same instance is tagged with different labels, resulting in incorrect classification. For example, as mentioned in the Methods section, the instances of C1-D2 and C2-D2 are the same but tagged with different labels. To solve this problem, entity pretreatment methods are presented.

**Figure 2:**
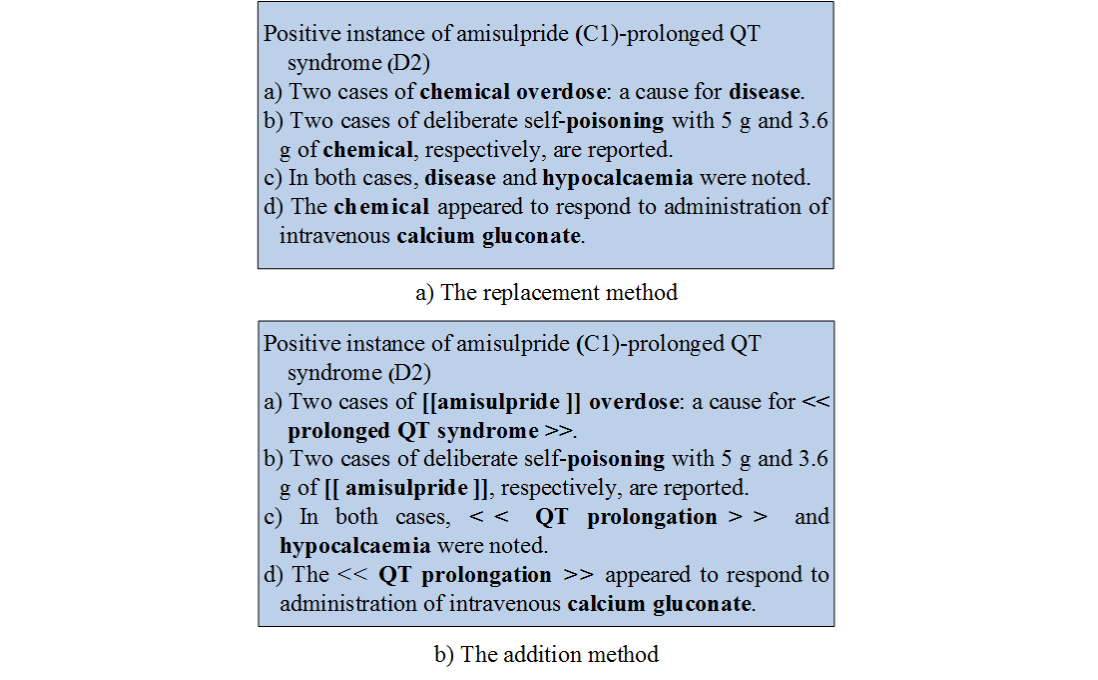
An instance with two different biomedical entity pretreatments.

There are 2 different biomedical entity pretreatments, as shown in Figure 2. In the first pretreatment, the target chemical and disease entities are respectively replaced with “chemical” and “disease,” which are called the replacement method. For example, in the instance of C1 and D2, sentence a) will be processed into “Two cases of chemical entity: a cause for disease.” In addition to the replacement method, there is another data preprocessing method, called the addition method. Different marks are added to the boundaries of chemical and disease entities, related to the relation instance. For instance, sentence a) will be processed into “Two cases of [[ amisulpride ]] overdose: a cause for << prolonged QT syndrome >>”. In the Results section, we will describe the advantages and disadvantages of the 2 different pretreatment methods.

### Model Architecture

**Figure 3:**
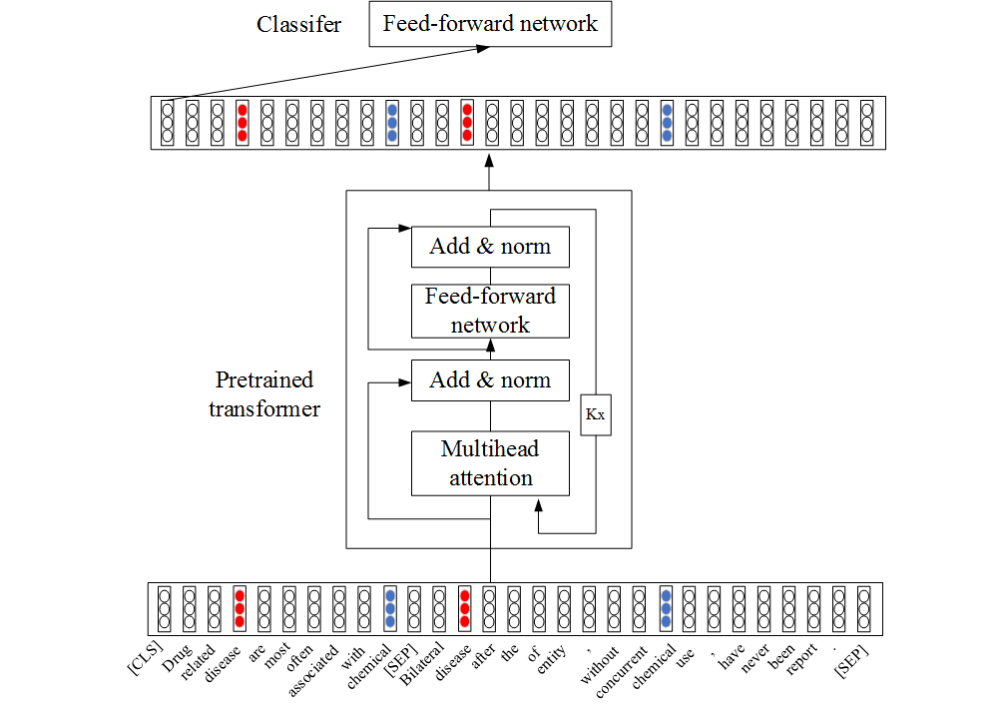
The architecture of the model.

As shown in Figure 3, when adopting this data preprocessing, the length of most instances is very long, which results from very long-distance dependencies and complex semantics.

Self-attention structure can directly calculate similarities between words, so that the distance between words is 1, which can intuitively solve long-distance dependencies. As demonstrated by Tang et al [21], Transformer, a combined self-attention structure, is capable of semantic feature extraction far exceeding that of RNN and CNN and performs better when increasing the number of attention structures. Therefore, a pretrained self-attention structure, namely a pretrained transformer, is applied for these problems.

However, for document-level relationship extraction, according to our preprocessing method, the length of instances is longer than the experimental data in the paper by Tang et al [21], and the semantics are more complicated. There are multiple target entity pairs in the instances; some reflect the correct relationship, and some do not. Therefore, the transformer structure must have a certain reasoning ability. To verify the validity of a pretrained self-attention structure on document-level relation extraction, we adopted the structure of SciBERT, which was pretrained on the scientific literature, and added a feed-forward network (FNN) as a classifier. A visual model architecture is provided in Figure 3. We fine-tuned the model on the preprocessed CDR dataset. The structure of model is described in detail in the following paragraphs.

Basde on the structure of BERT, SciBERT built a new vocabulary, called SCIVOCAB, and was trained on a scientific corpus that consists of computer science domain and biomedical papers. Following SciBERT, we still employ the same input representation, constructed by summation of token embedding, segment embedding, and position embedding. The tokens “[CLS]” and “[SEP]” are added at the beginning and end, respectively, of each instance. In addition, when tokenizing words, WordPiece embedding [23] was used with SCIVOCAB to separate words and split word pieces with “##”.

SciBERT is made up of N transformer stacks. Transformer stack *k* is denoted by *Transformer_k_*, which has its own parameters and consists of 2 components: multi-head attention and FFN.

Sk = Transformerk(Sk-1) (**1**)

where *S^k^* ∈ *R^n^*^×^*^d^* is the output of the transformer stack *k*. S^0^ is the input representation of text sequence *X, X* ∈*R^n^*^×^*^d^*. *n* is the length of text sequence, and *d* is the dimension of input representation. The whole text sequence shares the same parameters as the transformers.

The multihead applies self-attention, or scaled dot-product attention, multiple times. Through the mapping of the query *Q*, key *K*, and value *V*, scaled dot-product attention obtains a weighted sum of the values. *Q, K, V*∈ *R^n^*^×^*^d^* are the same matrices in the self-attention computation that are the input of transformer.

Instead of applying a single scaled dot-product attention, multihead attention applies query *Q*, key *K*, and value *V* to linearly project the input *h* times with different, learned linear projections to *n* x *l* dimensions, respectively, where *l = d/h* and *h* is the number of the head. The reason for that is multihead attention can form different representation subspaces at different positions, learn more semantic information, and capture long-distance dependencies better.

Oh = softmax(QWiQ(KWiK)T / sqrt(dh))VWiV (**2**)

Where the projections are parameter matrices *W^Q^* ∈ *R^d^*^×^*^l^*, *W^K^* ∈ *R^d^*^×^*^l^*, *W^V^* ∈ *R^d^*^×^*^l^*, and *O_h_* ∈ *R^d^*^x^*^l^.* *sqrt(d_h_)* is a scale factor to prevent the result of the dot-product attention from enlarging, and *sqrt(‧)* indicates that the square root is extracted.

Then, the outputs of the individual attention heads are merged, denoted as *O* ∈ *R^d^*^x^*^l^*. The input and output of the multihead attention are connected by residual connection. Layer normalization, denoted *LN*, is applied to the output of the residual connection.

*O =* [*o_1_;...;o_h_*] (**3**)

*M* = *LN*(*S^k-1^*+*O*) (**4**)

Where *M* ∈ *R^n^*^×^*^d^*

The second component of the transformer stack is 2 layers of pointwise FFN. On the other hand, it can be described as 2 convolutions with kernel size 1.

*S^k^ = ReLU*(*MW_1_*+*b_1_*)*W_2_*+*b_2_* (**5**)

where *W_1_* ∈ *R^d×m^, b_1_* ∈ *R^n×m^, W_2_* ∈ *R^m×d^,* and *b_2_* ∈ *R^n×d^*. Each row of or is the same, and *m* = *4d*.

The final layer is an FFN, a relation classifier. It corresponds to the final output of transformer of the token “[CLS]”.

*c = W^pred^s_1_* (**6**)

Where *W^pred^* ∈ *R^o×d^* is the weight matrix and s ∈ *R^d^* is the final output of the token “[CLS]”.

## Results

### Overview

In this section, we first describe some experimental datasets and provide some experiment settings. Then, we compare the performance of SciBERT with that of existing methods and validate the availability of the pretrained self-attention structure on the document-level dataset through the visualization of the multihead attention. Finally, experimenting on different datasets, including 2 sentence-level corpora and a document-level corpus, we compare various biomedical entity pretreatments and analyze which preprocessing is better for the self-attention structure.

### Datasets

Table 1 shows the statistics of the CDR [3], protein-protein interactions affected by mutations (PPIm) [24], DDI [1], and CPR [2] datasets. The CDR and PPIm datasets are document-level annotated corpus, and the DDI and CPR datasets are sentence-level annotated corpora, which are only used to discuss the advantages and disadvantages of different biomedical entity pretreatments.

**Table 1:** Descriptions of the chemical-disease relation datasets.

Dataset, Types		Training set	Development set	Test set
**CDR^a^**				
	Documents	500	500	500
	Positive	1038	1012	1066
	Negative	4324	4134	4374
**PPIm^b^**				
	Documents	597	N/A^c^	635
	Positive	750	N/A^c^	869
	Negative	1401	N/A^c^	1717
**DDI^d^**				
	Sentence	18,872	N/A^c^	3843
	Positive	3964	N/A^c^	970
	Negative	14,908	N/A^c^	2873
	Int	183	N/A^c^	96
	Advice	815	N/A^c^	219
	Effect	1654	N/A^c^	357
	Mechanism	1312	N/A^c^	298
**CPR^e^**				
	Sentences	6437	3558	5744
	Positive	4172	2427	3469
	Negative	2265	1131	2275
	CPR:3	777	552	667
	CPR:4	2260	1103	1667
	CPR:5	173	116	198
	CPR:6	235	199	293
	CPR:9	727	457	644

^a^CDR: chemical-disease relation.

^b^PPIm: protein-protein interaction affected by mutations.

^c^Development sets do not exist in the PPIm an dDDI datasets.

^d^DDI: drug-drug interaction.

^e^CPR: chemical-protein relation.

The CDR dataset is used to extract CID and is a 2-label classification task. The PPIm dataset is released to extract protein-protein interactions affected by genetic mutations, which is a 2-label classification. Aimed at extracting drug-drug interactions, DDI is concerned with classifying into 5 relation types, including the int type, advice type, effect type, mechanism type, and negative type. For the DDI dataset, we adopted some rules to filter some negative sentences as described by Quan et al [25]. With the purpose of extracting chemical-protein relations, the CPR dataset is labeled as 10 types of chemical-protein relations, 5 of which are used for evaluation. The chemical-protein relations of CPR are classified into 6 categories.

Due to the size of the CDR dataset, we merged the training and development sets to construct the training set. After preprocessing the CDR and PPIm datasets, we counted the average number of sentences per instance, average number of tokens per instance, and average number of tokens per sentence in the constructed instance set. Table 2 shows the statistics of the constructed instance set.

### Experiment Setup

We employed the parameters of the uncased SciBERT with the vocabulary SCIVOCAB and fine-tuned on the CDR datasets. The model parameters are described as: SciBERT_uncased_: *k* = 12, *h* = 12, *d* = 768, *m* = 3072.

Due to the distinction of the length of instances in the dataset, the input dimensions of the corresponding model for each dataset are different. For the CDR and PPIm datasets, the length of the input sequence is set to 512, and the batch size is set to 6. For the DDI dataset, the length of the input sequence is set to 150, and the batch size is set to 32. For the CPR dataset, the length of the input sequence is set to 200, and the batch size is set to 23. The epoch of all model training is set to 3. All results are averaged across 5 runs. For consistency of comparisons, we merged the training and development sets to train the models.

**Table 2:** Statistics of the constructed instance sets of the chemical-disease relation (CDR) and protein-protein interaction affected by mutations (PPIm) datasets.

Dataset, Types		Training set	Test set
**CDR with preprocessing**			
	Instances	10,407	5418
	Positive	1947	1042
	Negative	8460	4376
	Sentences per instance	11.1	12.1
	Tokens per instance	161.5	168.9
	Tokens per sentence	14.6	14.0
**PPIm with preprocessing**			
	Instances	2151	2586
	Positive	750	869
	Negative	1401	1717
	Sentences per instance	9.0	8.8
	Tokens per instance	169.6	186.6
	Tokens per sentence	18.7	21.2

### Comparison of the Pretrained Self-Attention Structure With Other Methods

For the CDR dataset, we compared our method with 6 state-of-the-art models without any knowledge bases. Zhou et al [9] proposed a method based on feature engineering and long short-term memory. Gu et al [8] combined CNN with maximum entropy. A recurrent piecewise CNN [13] was the piecewise CNN. A bi-affine relation attention network [14] incorporated an attention network, multi-instance learning, and multitask learning. A labeled edge graph CNN [12] was used for document-level dependency graphs. For the PPIm dataset, we compared our method with 4 models. Because few studies focused on the PPIm dataset, the 4 models are not really state-of-the-art. Table 3 shows the result of the comparisons.

As shown in Table 3, compared with other approaches, our method with the replacement method greatly improved the precision. The F1 score is 1.9% higher than the best result from Vargas et al [19] with the CDR test set. Our method also has great performance with the PPIm. It shows that a pretrained self-attention structure can be suitable for a document-level dataset.

**Table 3:** Performance of the chemical-disease relation (CDR) and protein-protein interactions affected by mutations (PPIm) test datasets compared with state-of-the-art methods.

Dataset, Model		P^a^, %	R^b^, %	F1, %
**CDR**				
	LSTM^c^ [9]	55.6	68.4	61.3
	CNN^d^ [8]	55.7	68.1	61.3
	RPCNN^e^ [13]	55.2	63.6	59.1
	BRAN^f^ [14]	55.6	70.8	62.1
	GCNN^g^ [12]	52.8	66.0	58.6
	Our method	65.5	62.6	64.0
**PPIm**				
	SVM^h^ [26]	32.0	34.0	33.0
	CNN (without KB^i^) [27]	38.2	37.3	37.8
	MNM^j^ [28]	40.3	32.3	35.9
	MNM+Rule [28]	38.0	37.0	37.5
	Our method	83.5	90.4	86.8

^a^P: precision.

^b^CNN: convolutional neural network.

^c^R: recall.

^d^LSTM: long short-term memory.

^e^RPCNN: recurrent piecewise convolutional neural network.

^f^BRAN: bi-affine relation attention network.

^g^GCNN: graph convolutional neural network.

^h^SVM: support vector machine.

^i^KB: knowledge base.

^j^MNM: memory neural network.

### Effects of Pretreatment Methods for Biomedical Entities

As described later, there are 2 methods, one of which is the replacement method, that replace biomedical entities with uniform words. The second method is the addition method, which adds extra tags in the left and right sides of biomedical entities. We conducted experiments with the CDR, PPIm, DDI, and CPR datasets. The comparison of the 2 pretreatments for biomedical entities is shown in Table 4.

For each dataset, the recall rate and F1 score obtained with our model with the replacement method were higher than obtained with our model with the addition method, especially for the CDR dataset. The reason is that biomedical entities are complicated, and most are compound words. For the pretrained self-attention structure, the word embeddings of biomedical entities are hard to learn from small biomedical datasets. As a consequence, replacing the target entities with uniform words is beneficial for the model to understand target entities and pay more attention in the context of target entities.

**Table 4:** Comparison of 2 pretreatments (addition and replacement) for biomedical entities using our method.

Dataset, Types		Addition method			Replacement method		
		P^a^, %	R^b^, %	F1, %	P, %	R, %	F1, %
**CDR^c^**							
	Positive	67.4	54.8	60.4	65.5	62.6	64.0
**PPIm^d^**							
	Positive	79.3	91.5	84.8	83.5	90.4	86.8
**DDI^e^**							
	Int	74.8	46.2	57.1	76.2	46.9	58.0
	Advise	87.2	84.7	85.9	88.6	89.0	88.8
	Effect	77.2	82.1	79.5	77.0	82.6	79.7
	Mechanism	84.8	80.4	82.5	82.1	86.0	84.0
	All	81.6	78.6	80.0	81.2	81.3	81.4
**CPR^f^**							
	CPR:3	73.5	80.3	76.7	75.4	79.5	77.4
	CPR:4	84.4	88.8	86.6	83.7	90.4	86.9
	CPR:5	80.7	82.0	81.3	81.2	86.5	83.7
	CPR:6	84.0	89.4	86.7	86.5	88.2	87.3
	CPR:9	76.2	86.9	81.2	79.5	90.1	84.5
	All	80.4	86.5	83.3	81.4	87.9	84.5

^a^P: precision.

^b^R: recall.

^c^CDR: chemical-drug reaction.

^d^PPIm: protein-protein interaction affected by mutations.

^e^DDI: drug-drug interaction.

^f^CPR: chemical-protein reaction.

### Comparison of Different Pretrained Models

BERT and SciBERT are the pretrained models that have the same self-attention structure. The difference between the two is that BERT is pretrained on the wiki corpus and SciBERT is pretrained on a large quantity of scientific papers from the computer science and biomedical domains. Table 5 presents the comparison of BERT and SciBERT on 4 biomedical data sets. As shown by Table 5, SciBERT performs better than BERT, particularly with the F1 score, which was improved by 3.5% on the CDR data set. Therefore, the model pretrained on the biomedical corpus is beneficial for extracting biomedical relations.

**Table 5:** Comparison of different pretrained models using our method.

Dataset, Type			BERT						SciBERT				
		P^a^, %		R^b^, %		F1, %		P, %		R, %		F1, %	
**CDR^c^**													
	Positive		62.9		58.3		60.5		65.5		62.6		64.0
**PPIm^d^**													
	Positive		79.0		92.2		85.1		83.5		90.4		86.8
**DDI^e^**													
	Int		69.8		42.7		52.8		76.2		46.9		58.0
	Advise		91.3		89.0		90.1		88.6		89.0		88.8
	Effect		74.1		77.6		75.7		77.0		82.6		79.7
	Mechanism		78.5		80.1		79.3		82.1		86.0		84.0
	All		79.0		77.5		78.2		81.2		81.3		81.4
**CPR^f^**													
	CPR:3		73.8		76.5		75.1		75.4		79.5		77.4
	CPR:4		81.7		89.7		85.5		83.7		90.4		86.9
	CPR:5		79.3		80.7		79.9		81.2		86.5		83.7
	CPR:6		80.2		84.6		82.2		86.5		88.2		87.3
	CPR:9		76.5		88.2		81.9		79.5		90.1		84.5
	All		79.0		85.9		82.3		81.4		87.9		84.5

^a^P: precision.

^b^R: recall.

^c^CDR: chemical-drug reaction.

^d^PPIm: protein-protein interaction affected by mutations.

^e^DDI: drug-drug interaction.

^f^CPR: chemical-protein reaction.

### Analysis of Each Component of the Method

Data preprocessing (DP) and pretraining means (PTM) are important components of our method; DP aims to alleviate noise, and PTM is designed to solve the long-distance dependencies. We compared the importance of each component of our method with the CDR dataset. Table 6 shows the changes in performance on the CDR dataset by removing DP and PRM. PTM resulted in a greater performance improvement than DP.

**Table 6:** Performance changes by removing different parts of our model.

CDR^a^ dataset	P^b^, %	R^c^, %	F1, %	Change, %
Baseline	65.5	62.6	64.0	N/A
Remove DP^d^	67.0	54.3	60.0	–6.3
Remove PTM^e^	46.1	39.5	42.6	–33.4
Remove DP and PTM	48.9	31.2	38.1	–40.5

^a^CDR: chemical-drug reaction.

^b^P: precision.

^c^R: recall.

^d^DP: data preprocessing.

^e^PTM: pertraining means.

## Discussion

### Principal Findings

To fully illustrate that our model can solve the problem of long-distance dependencies, we set 50 as the unit of instance length to count the number of positive and negative instances of the CDR test set, as shown in Table 7. As can be seen from the table, the instance length of the test sets is concentrated in the range of 50 to 300.

We calculated the precision rate, recall rate, and accuracy rate of each interval length in the test set. The results are shown in Table 8. As can be seen in the table, the model has good performance when the instance length is longer than 100, except for the instances with lengths of 201 to 250. Therefore, our model can capture long-distance dependencies.

**Table 7:** Quantity distribution of the chemical-disease relation test set.

Interval length	Positive	Negative	Sum
0-50	70	344	414
51-100	181	884	1065
101-150	200	845	1045
151-200	160	756	916
201-250	158	663	821
251-300	177	571	748
301-350	56	216	272
351-400	34	64	98
>400	6	33	39

**Table 8:** Results of each interval length in the test set using our replacement method.

Interval length	P^a^, %	R^b^, %	F1, %
0-50	54.2	64.3	58.8
51-100	57.5	57.5	57.5
101-150	67.7	64.0	65.8
151-200	64.4	71.2	67.7
201-250	66.2	54.4	59.7
251-300	69.9	69.5	69.7
301-350	70.8	60.7	65.4
351-400	80.0	82.4	81.2
>400	100.0	66.7	80.0

^a^P: precision.

^b^R: recall.

**Figure 4:**
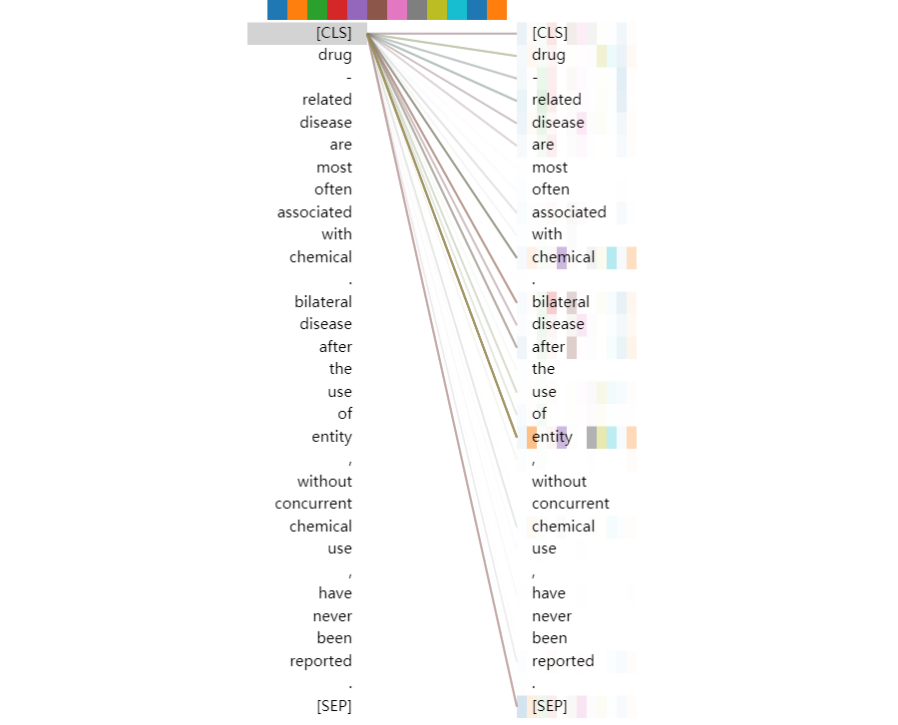
Visualization of the output of token “[CLS]” in the multihead attention of the final transformer stack.

To verify that the pretrained self-attention mechanism works as we believe, which is that it can take advantage of the textual context and capture very long-range dependencies to understand the complex semantics of biomedical text, we visualized the output of token “[CLS]” in the multihead of the final transformer stack, as shown in Figure 4.

As seen by the token colors, the token “[CLS]” is related to the following tokens: “chemical,” “disease,” “drug,” “related,” “bilateral,” “[CLS],” and “[SEP]”. The 12 different colors refer to the different head attentions. The more and darker the colors, the more relevant the token. Lines between two tokens denote a correlation between two tokens. Their clarity depends on the result of the head attentions.

From the perspective of semantic analysis, there are 2 places in this example reflecting a relationship between a disease and chemical: “Drug-related disease are most often associated with chemical.” and “Bilateral disease after the use of entity, without concurrent chemical use, have never been reported.” In the first sentence, the relation between chemical and disease is mainly determined by the following tokens: “associated,” “chemical,” “disease,” “related,” and “drug.” In the second sentence, the relation between chemical and disease is mainly determined by the following tokens: “reported,” “never,” “chemical,” “disease,” “without,” and “concurrent.” Token “[CLS]” is related to the most keywords in both sentences. Therefore, the pretrained self-attention structure can take advantage of the textual context and capture very long-range dependencies from document-level instances. On the other hand, the distribution of the different colors shows that multihead attention can form diverse representation subspaces to learn more complicated semantics.

**Figure 5:**
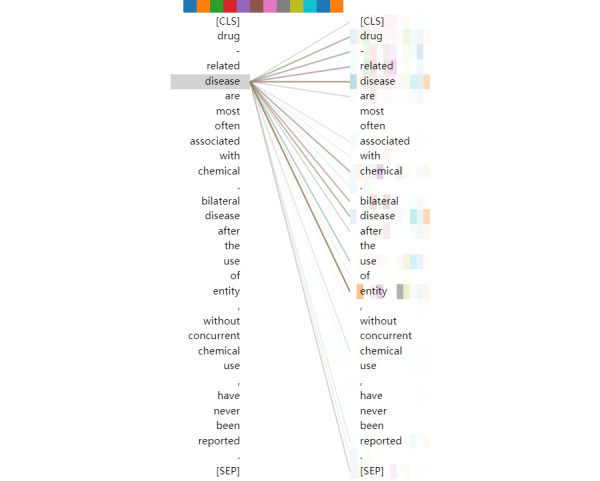
Visualization of the output of token “disease” in the final multihead attention.

**Figure 6:**
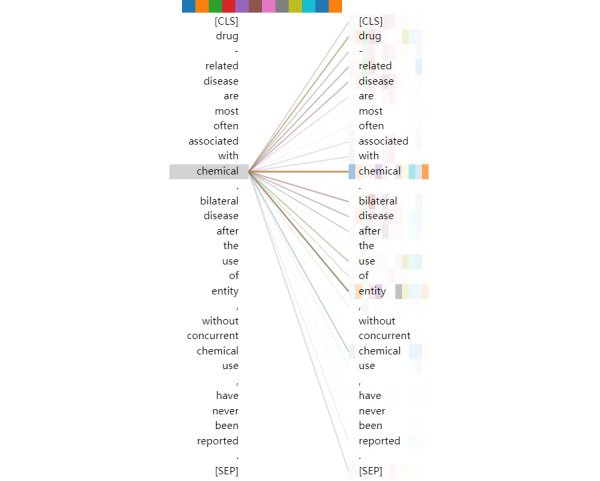
Visualization of the output of token “chemical” in the final multihead attention.

However, from the gradation of the colors, the relationship between token “[CLS]” and the keywords is not strong enough. Token “[CLS]” is not highly correlated with token “disease” in this instance. We visualized the output of tokens “chemical” and “disease” in the final multihead attention, as shown in Figures 5 and 6. As seen in these figures, the tokens “chemical” and “disease” in the sentences capture more local information, compared with the token “[CLS].” It may be inferred that, for document-level relation extraction in the final layer of the pretrained self-attention structure, designing a special network to capture the relationships between different target entities is better than applying a dense layer.

### Conclusions

For a document-level annotated dataset, instead of dividing the dataset, we considered all target entity pairs as a whole and applied a pretrained self-attention structure to extract biomedical relations. The results and analysis show that the pretrained self-attention structure extracted relations of multiple entity pairs in a document. Through the visualization of the transformer, we verified that the pretrained self-attention structure can capture long-distance dependencies and learn complicated semantics. Furthermore, we conclude that replacement of biomedical entities benefits biomedical relation extraction, especially for document-level relation extraction.

However, this method still has some issues. In future work, we plan to design a more effective network to capture local relations between biomedical entities and improve our method.

## Abbreviations

BRAN: bi-affine relation attention network.
CDR: chemical-disease relation.
CID: chemical-induced disease.
CNN: convolutional neural network.
CPR: chemical-protein relation.
DDI: drug-drug interaction.
DP: data preprocessing.
FNN: feed-forward network.
GCNN: graph convolutional neural network.
KB: knowledge base.
MNM: memory neural network.
P: precision.
PPIm: protein-protein interactions affected by mutations.
PTM: pretraining means.
R: recall.
RNN: recurrent neural network.
RPCNN: recurrent piecewise convolutional neural network.
SVM: support vector machine.
